# The dose-response analysis between BMI and common chronic diseases in northeast China

**DOI:** 10.1038/s41598-018-22551-y

**Published:** 2018-03-09

**Authors:** Jianxing Yu, Yuchun Tao, Jing Dou, Junsen Ye, Yaqin Yu, Lina Jin

**Affiliations:** Epidemiology and Biostatistics, School of Public Health, No. 1163 Xinmin Street, Jilin University, Changchun, Jilin, 130021 China

## Abstract

High body mass index (BMI) predisposes to several chronic diseases, but a large-scale systematic and detailed study of dose-response relationship between BMI and chronic diseases has not been reported previously. In this study, we aimed to investigate the relationship between BMI and 3 chronic diseases (hypertension, dyslipidemia and MetS) in northeast China. A sample of 16412 participants aged 18~79 years old were included in Jilin province in 2012. The lambda-mu-sigma (LMS) method was applied to examine the trend of BMI by age, and the restricted cubic splines were used to investigate the non-linear associations (dose-response curve) between BMI and chronic diseases. It was pointed out that BMI increased rapidly when young, then kept steady in middle age, and finally declined slowly in old age, and accordingly age was divided into 3 segments, which were different by gender. The odds ratios (ORs) of BMI for the chronic diseases increased relatively slowly when young, then increased dramatically in middle-age and old population, especially for men. Further, the ORs of BMI among non-smokers were lower than those among smokers, and the same trend was shown to be more apparent among drinkers and non-drinkers. The risk of BMI for common chronic diseases increased dramatically in middle-aged, especially for men with drinking and smoking habits.

## Introduction

Obesity is believed as a major risk factor for common chronic diseases, like hypertension, dyslipidemia, diabetes and metabolic syndrome (MetS)^1,2^. And the prevalence of obesity is developing extremely fast worldwide nowadays, especially in China^3,4^. Therefore, understanding the effects of obesity on chronic diseases has never been more urgent, given the rapid rise in obesity worldwide in recent years^5,6^.

Many obesity indices have been used in the literatures, such as body mass index (BMI), waist circumference (WC) and waist-hip ratio, which have measured the obesity from different angles and in different aims. Whereas, a lot of studies have pointed out that BMI is not only a good predictor in the chronic disease studies^7–10^, but also has more classification (“Underweight”, “Normal weight”, “Overweight” and “Obese”) compared with other indices^11–14^. Therefore, BMI has become one of the most frequently used indices for obesity in the chronic disease studies.

However, it also has brought great limitations, because BMI is treated as a categorical variable in most studies. On one hand, it is illogical that the OR of the “Overweight” (with BMI from 25 to 30 kg/m^2^) was 2.03, while the OR rocketed to 6.11 for “Obese” (with BMI ≥30 kg/m^2^)^8^, especially those with BMI around 30 kg/m^2^. The comparison of the results from different populations is a little complex and hard to be standardized, due to the inconsistent cut-offs. On the other hand, the development of obesity is a continuous and long-term process, the categorical variable was less powerful and less sensitive in measuring the extent of obesity in its progress. Therefore, a continuous BMI is believed to be better than a categorical one in evaluating the obesity.

Fortunately, the dose-response curve can provide the continuous ORs^15^, especially for the non-linear associations between BMI and chronic diseases. In addition, the restricted cubic splines is one of the most ideal function models used in dose-response analysis, which allows the risk to vary without sudden jump from one interval to the next one^16^. In this study, we aimed to investigate the continuous ORs of BMI on three common chronic diseases (hypertension, dyslipidemia and MetS) in Jilin province in 2012. The BMI-chronic disease associations were analyzed stratified by gender, age, smoking and drinking status. We found that the ORs of BMI for 3 chronic diseases showed a nonlinear increased tendency with BMI, and the curves were different among the diseases.

## Results

### Descriptive characteristics of the participants by age

**Figure 1:**
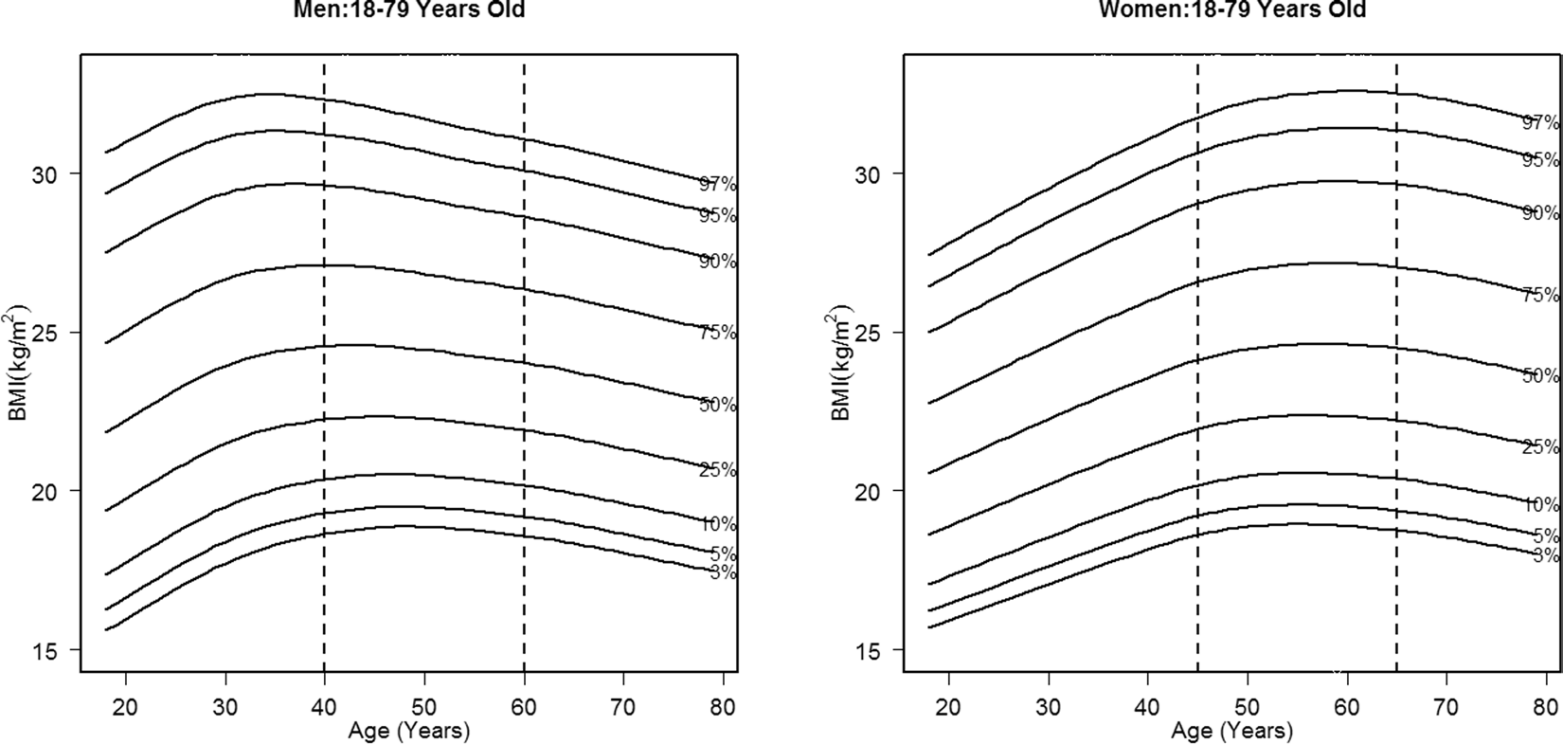
BMI percentages by age for men and women (n = 16,412) in Jilin in 2012. The curve represents BMI percentage.

Figure 1 shows the smoothed BMI percentile curves by age for males and females. In males, BMI increased from 18 to 39 years old, then kept steady from 40 to 59 years old, and declined slowly after 60 years old. While in females, the BMI increased rapidly from 18 to 44 years old, then relatively stable from 45 to 64 years old, and finally decreased after 65 years old. Thus, 18~39, 40~59 and 60~79 can be viewed as BMI rising (Young), stable (Middle-age) and decline (Old) age groups for men, and 18~44, 45~64 and 65~79 were corresponding age groups for women.

The descriptive characteristics of the participants were shown in Table 1. The differences for all variables under consideration among age groups were statistically significant, where BMI and the prevalence of common chronic diseases were associated with age. Further, it was implied that the prevalence of common chronic diseases increased with age, which was different from the trend of BMI by age, so the subsequent analyses were listed respectively by age groups.

**Table 1:** Descriptive characteristics of the participants by age (n = 16,412) in Jilin in 2012 [$\overline{X}\pm S$/n (%)].

Variable	Total	Young (18~)	Middle-age (40~/45~)	Old (60~79/65~79)	*t/χ* ^2^	*P* value
Men						
BMI(kg/m^2^)	24.34 ± 3.62	24.09 ± 4.04	24.62 ± 3.39	23.96 ± 3.43	25.10	<0.001
Hypertension	3041(40.45)	448(19.67)	1749(45.81)	844(59.39)	665.73	<0.001
Dyslipidemia	3324(44.22)	918(40.30)	1844(48.30)	562(39.55)	52.50	<0.001
Diabetes	789(10.50)	65(2.85)	477(12.49)	247(17.38)	229.57	<0.001
MetS	2575(34.26)	573(25.15)	1536(40.23)	466(32.79)	145.67	<0.001
Smoking	3950(52.55)	1278(56.37)	2120(55.53)	546(38.42)	140.59	<0.001
Drinking	4316(57.42)	1371(60.18)	2354(61.66)	591(41.59)	180.76	<0.001
Women						
BMI(kg/m^2^)	24.18 ± 3.66	23.27 ± 3.65	24.79 ± 3.53	24.64 ± 3.61	183.14	<0.001
Hypertension	2980(33.50)	416(12.05)	1986(43.45)	578(66.36)	1338.52	<0.001
Dyslipidemia	3176(35.71)	661(19.14)	2039(44.61)	476(54.65)	706.57	<0.001
Diabetes	829(9.32)	85(2.46)	576(12.60)	168(19.29)	352.82	<0.001
MetS	2806(31.55)	516(14.94)	1871(40.93)	419(48.11)	737.84	<0.001
Smoking	1013(11.39)	220(6.37)	654(14.31)	139(15.96)	142.76	<0.001
Drinking	843(9.48)	457(13.23)	353(7.72)	33(3.79)	106.09	<0.001

### Association between BMI and chronic diseases with 95% CI

**Figure 2:**
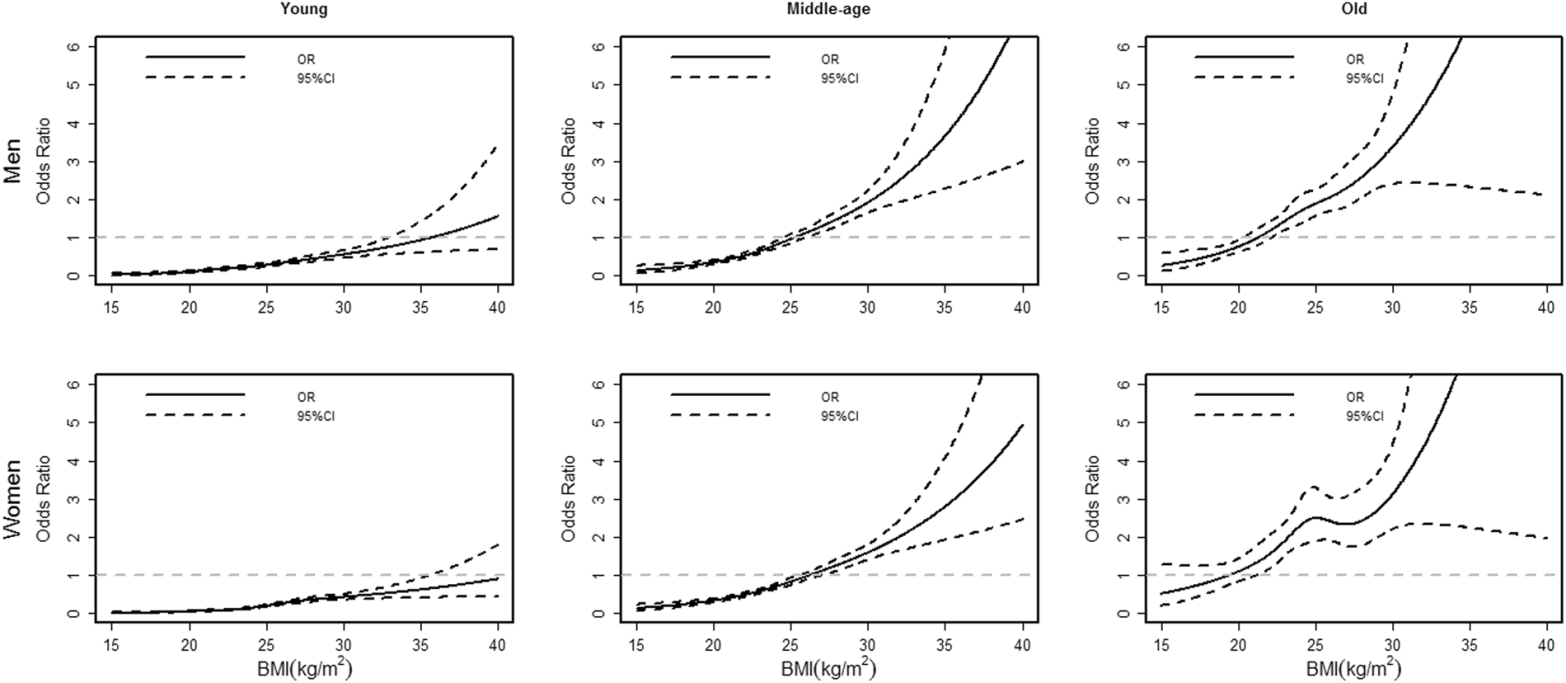
Association between BMI and hypertension with 95% CI (n = 16,412) in Jilin in 2012. The solid line represents OR, and the dotted line represents 95% CI.

Figure 2 shows how the ORs of BMI developed in hypertension by gender and age. Generally, the ORs increased with BMI in hypertension, and for both males and females under different age groups. Further, the ORs of BMI increased relatively slowly in young group, and accelerated in middle-age and old groups. The dose-response curves for dyslipidemia and MetS showed the similar trend (see details in part 3 of the supplementary file), but the results for diabetes were not included, due to the relatively lower prevalence, which may cause unreliable tendency. Given the same BMI, the ORs of BMI were larger in old group than those in other age groups. Moreover, the 95% CIs of BMI beyond 18~30 were a little wider, due to that less than 10% participants were with BMI < 18 or BMI > 30, that was, the tendency of BMI within the range 18~30 was relatively reliable and stable.

### Comparison of the BMI-chronic diseases associations by gender

**Figure 3:**
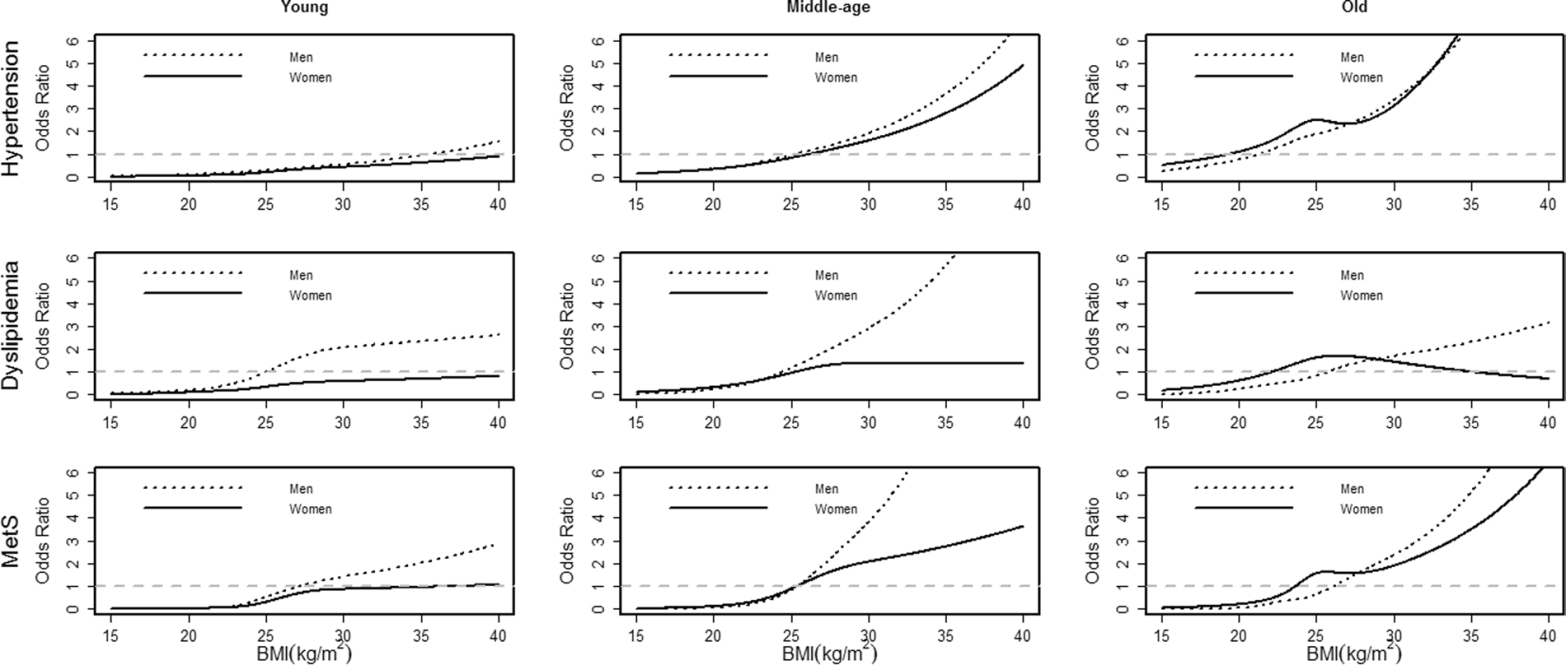
Comparison of the BMI-chronic diseases associations between men and women (n = 16,412) in Jilin in 2012. The solid line represents women, and the dotted line represents men.

The trends for ORs of BMI were similar among chronic diseases by gender, except dyslipidemia. The associations between BMI and chronic diseases were more marked in men than women in most cases (Fig. 3), and the ORs of higher BMI in males were extremely greater than those in females in dyslipidemia. Moreover, the ORs of BMI among dyslipidemia in females were fairly low as BMI increased. It should be pointed out that the gender gap in ORs of BMI peaked in the middle-aged.

### Comparison of the BMI-chronic diseases associations by smoking and drinking status

**Figure 4:**
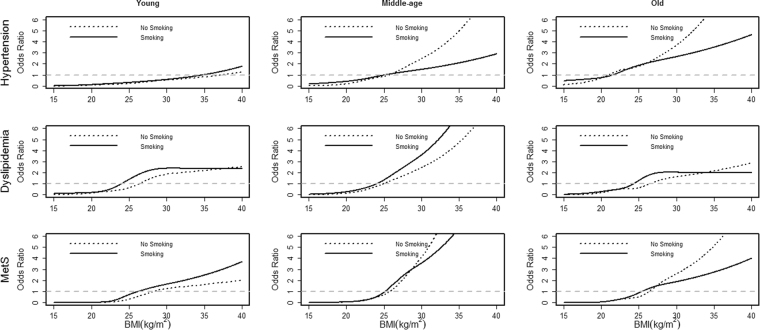
Comparison of the BMI-chronic diseases associations by smoking status for males (n = 7517) in Jilin in 2012. The solid line represents smoking, and the dotted line represents no smoking.

**Figure 5:**
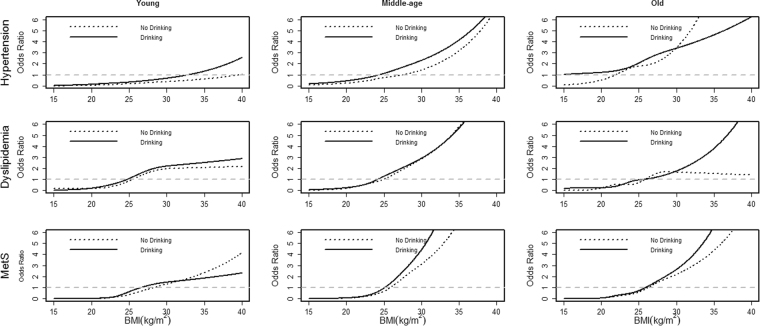
Comparison of the BMI-chronic diseases associations by drinking status for males (n = 7517) in Jilin in 2012. The solid line represents drinking, and the dotted line represents no drinking.

Finally, smoking and drinking status were investigated, and Table 2 shows the prevalence of the chronic diseases under different smoking and drinking status, respectively. Similarly, Figs 4 and 5 shows how the ORs of BMI developed in hypertension, dyslipidemia and MetS for males under different smoking and drinking status, respectively (the results for women were not included, due to the relatively low prevalence of smoking and drinking, which may cause unreliable tendency). Generally, the trends of ORs of BMI were similar for either smoking or drinking, especially among young participants, and the ORs of BMI in the middle-age population with smoking or drinking habits had obviously increased. Meanwhile, the ORs of BMI among non-smokers were a little lower than those among smokers, but such analysis towards drinking status was different, that is, the ORs of BMI among drinkers were almost uniformly higher than those among non-drinkers.

**Table 2:** Prevalence of chronic diseases by smoking & drinking status in Jilin in 2012 [n (%)].

Variable	Male (n = 7517)		*χ* ^*2*^	*P*-value	Female (n = 8895)		*χ* ^*2*^	*P*-value
	Yes	No			Yes	No		
Smoking								
Hypertension			17.14	<0.001			4.39	0.036
Yes	1510(38.23)	1531(42.92)			369(36.43)	2611(33.13)		
No	2440(61.77)	2036(57.08)			644(63.57)	5271(66.87)		
Dyslipidemia			7.87	0.005			36.99	<0.001
Yes	1807(45.75)	1517(42.53)			449(44.32)	2727(34.6)		
No	2143(54.25)	2050(57.47)			564(55.68)	5155(65.4)		
Diabetes			23.70	<0.001			9.32	0.002
Yes	350(8.86)	439(12.31)			121(11.94)	708(8.98)		
No	3600(91.14)	3128(87.69)			892(88.06)	7174(91.02)		
MetS			8.27	0.004			14.73	<0.001
Yes	1294(32.76)	1281(35.91)			373(36.82)	2433(30.87)		
No	2656(67.24)	2286(64.09)			640(63.18)	5449(69.13)		
Drinking								
Hypertension			44.25	<0.001			27.54	<0.001
Yes	1886(43.70)	1155(36.08)			214(25.39)	2766(34.35)		
No	2430(56.30)	2046(63.92)			629(74.61)	5286(65.65)		
Dyslipidemia			14.29	<0.001			8.68	0.003
Yes	1989(46.08)	1335(41.71)			262(31.08)	2914(36.19)		
No	2327(53.92)	1866(58.29)			581(68.92)	5138(63.81)		
Diabetes			3.63	0.060			26.79	<0.001
Yes	428(9.92)	361(11.28)			37(4.39)	792(9.84)		
No	3888(90.08)	2840(88.72)			806(95.61)	7260(90.16)		
MetS			28.98	<0.001			20.37	<0.001
Yes	1588(36.79)	987(30.83)			208(24.67)	2598(32.27)		
No	2728(63.21)	2214(68.17)			635(75.33)	5454(67.73)

## Discussion

In this study, dose-repose curve was applied to provide the continuous ORs of BMI on hypertension, dyslipidemia and MetS in Jilin province in 2012. It was pointed out that the ORs of BMI for hypertension, dyslipidemia and MetS increased with BMI, and the ORs speeded up since middle age. The ORs of BMI for chronic diseases were usually more marked in men than those in women, especially for dyslipidemia. Moreover, the ORs of BMI among men with drinking and/or smoking habits were generally higher than those without these habits, and the differences were even more apparent in the analysis of drinking status.

Firstly, age is believed as an influencing factor for both BMI and the chronic diseases, which was divided into 3 segments in this study according to the smoothed BMI percentile curves. Women were 5 years older than men in different segments, which was consistent with the fact that the life expectancy of women was 5 years older than men^17^. And other studies also pointed out that BMI increased faster among young participants than the middle-aged group, and the rate of increase slowed down among old-aged group^14^. Further, men were more likely to be overweight than women in the young group, which was consistent with other studies^18,19^.

Compared with previous analyses^7–9^, the dose-response analysis we have employed is more appropriate, because the continuous ORs are more accurate, and the dose-response curve is better to reflect the overall trend for the ORs of BMI. It was found that the ORs of BMI for hypertension, dyslipidemia and MetS increased with BMI, and they had accelerating trend to go up in middle-aged. Besides, previous studies also have documented that the people aged 40–60 were more likely to suffer from chronic diseases^20^, and we provided a new insight from the perspective of graphical. Further, the basal metabolic rate of middle-aged people were lower than that of young people^21,22^, while the body functioning of young people were better than that of middle-aged, which might cause the ORs of BMI for chronic diseases among middle-aged were larger those among young people.

The other thing needs to be noticed is that the ORs of BMI for all 3 common chronic diseases were usually more marked in men than women, especially in dyslipidemia. The reason that men tended to have larger ORs of BMI for chronic diseases might be ascribed to the great differences in the diet and habits between genders, for example, men tended to smoke, drink (Tables 1 and 3) and eat more meat^23,24^. Another possible explanation was the differences in body function, e.g., estrogen was believed to play an important role in fat metabolism regulation, and to restrain fat accumulated in waist^25–27^. This might be the reason for such significant difference in dyslipidemia between males and females, since estrogen might help females to decline the risk of BMI towards dyslipidemia.

**Table 3:** Reported smoking and drinking rates in China by the National Health and Family Planning Commission (NHFP).

Years	Smoking rates (%)			Drinking rates (%)		
	Total	Men	Women	Total	Men	Women
1993	32.0	59.3	5.0	—	—	—
1998	28.9	53.4	4.0	—	—	—
2003	26.0	48.9	3.2	8.2	15.3	1.1
2008	25.1	48.0	2.6	8.6	16.1	1.2
2013	—	—	—	14.7	28.0	2.0

Furthermore, the rates of smoking and drinking were extremely high in Jilin province^28^, compared with the Chinese average rates^29–31^. And some studies also suggested that smoking/drinking was tightly linked with both obesity and chronic diseases^32–34^. Most researchers focused on young and elderly smokers rather than the middle-aged smokers^35,36^, while we found that the middle-aged smokers had the higher ORs of BMI for chronic diseases. One possible reason was that the middle-aged people suffer from decline in organ function^21^. Moreover, Prabhat *et al*.^37^ suggested that the risk of diseases was decreased if quitting smoking happens before the age of 40.

Many researches pointed out that alcohol consumption is a major contributor to the prevalence of chronic diseases^30,38,39^, which was consistent with our study. Moreover, a report from NHFPC (National Health and Family Planning Commission) indicated that the drinking rates in China had a rising tendency (Table 3) these years^30,31^, while the smoking rates had an expected downward trend. What’s more, the excessive consumption of alcohol had been paid more attention to, and the relevant policies had been adopted in most developed countries, such as USA and UK^40–43^. Whereas, there is no relevant laws or regulations to curb excessive consumption of alcohol in China. Therefore, controlling or stopping excessive consumption of alcohol became particularly urgent, especially for men with large BMI.

Some limitations of our studies should be pointed out here. Firstly, the smoking and drinking status of the participants were self-report, which might be subject to reporting bias. Secondly, gender, age, smoking and drinking statuses were investigated in our study; however, other confounders which might have impacts on these chronic diseases, such as physical activity, diet and genes, were not under our consideration this time, which might have some slight effects on our results. Finally, the results were conducted from a cross-sectional study in Jilin province, which might limit our ability to generalize the results.

## Conclusions

The ORs of BMI for hypertension, dyslipidemia and MetS increased with BMI, and the risk of BMI had shown an accelerating trend to go up in middle-aged. The ORs of BMI among men with drinking and/or smoking habits were generally higher than those without these habits, and the differences were even more apparent in the analysis of drinking status.

## Methods

### Study population

Data were derived from a cross-sectional survey in Jilin Province of China in 2012. A total of 23050 participants who had lived in Jilin Province for more than 6 months and were 18–79 years old were selected through multistage stratified random cluster sampling^44^ (see details in Part 1 of the Supplementary Material online). For the purpose of the present analyses, some subjects were excluded due to missing values (6175 subjects) and the other subjects were excluded due to the fact that they were limited to do independent activities (463 subjects, who were paralyzed, disabled and other diseases that led to inactivity). Finally, a total of 16412 subjects were included in the present analyses.

### Data measurement

Height (cm) was measured to the nearest 0.5 cm using a portable stadiometer against the wall, with participants standing in an upright position on a flat surface without shoes, with the back of the heels and the occiput on the stadiometer. Weight (kg) was measured to the nearest 0.1 kg, using an electronic digital scale. BMI was calculated by dividing weight in kilograms by height in meters squared. Blood pressure was measured using mercury sphygmomanometer in the sitting position after a 10-min rest period by trained professionals. Two readings each of SBP and DBP were recorded, and the average of each measurement was used for data analysis. If the first two measurements differed by more than 5 mmHg, additional two readings were taken. Fasting glucose and serum lipids were measured in plasma samples by using a fingertip blood glucose monitor (Bayer, Leverkusen, Germany) and a MODULE P800 biochemical analysis machine (Roche Co., Ltd., Shanghai, China) (see details in Part 2 of the Supplementary Material).

### Assessment criteria

Hypertension was referred to those with systolic blood pressure (SBP) ≥140 mm Hg, diastolic blood pressure (DBP) ≥90 mm Hg, and/or the use of antihypertensive medications over the past 2 weeks^45^. Dyslipidemia was based on triglyceride (TG) ≥1.7 mmol/L, total cholesterol (TC) ≥5.2 mmol/L, high-density lipoprotein cholesterol (HDL-C) < 1.0 mmol/L, low-density lipoprotein cholesterol (LDL-C) ≥3.4 mmol/L^46^. Diabetes was defined as the use of hypoglycemic agents over the past 2 weeks or a self-reported history of diabetes or FBG ≥7.0 mmol/L^47^. MetS^48,49^ was defined as three or more of the following conditions clustered in one subject: a) WC ≥85 cm for males or ≥80 cm for females; b) TG ≥1.7 mmol/L; c) HDL-C < 1.00 mmol/L for males or <1.30 mmol/L for females; d) SBP ≥130 mmHg and DBP ≥85 mmHg, or ongoing antihypertensive drug therapy; and e) FBG ≥5.6 mmol/L or ongoing anti-diabetic drug treatment. Smoking was defined as having smoked at least one cigarette per day and more than 100 cigarettes in total over the past 30 days. Participants who drank alcoholic beverages at least once a week were characterized as drinkers.

### Statistical analysis

The continuous variables were expressed as means ± standard deviations (SD) and compared using the *t* test. The categorical variables were expressed as counts or percentages and compared using the Rao-Scott-*χ*^2^ test. The LMS method in the VGAM package was used to determine the trends of BMI by age, and the restricted cubic splines in the Hmisc package were used to investigate the dose-response curve of BMI-chronic diseases associations. All statistical analyses were performed with R version 3.3.2 (University of Auckland, Oakland, New Zealand). Statistical significance was set at a *P* value < 0.05.

### About the data

The survey was implemented by School of Public Health, Jilin University and Jilin Center for Disease Control and Prevention in Jilin Province in 2012. According to relevant regulations, we were sorry that the data can’t be shared.

## Electronic supplementary material

Supplementary Information
